# Parental responsiveness and children’s trait epistemic curiosity

**DOI:** 10.3389/fpsyg.2022.1075489

**Published:** 2023-01-26

**Authors:** Shoko Iwasaki, Yusuke Moriguchi, Kaoru Sekiyama

**Affiliations:** ^1^Graduate School of Advanced Integrated Studies in Human Survivability, Kyoto University, Kyoto, Japan; ^2^Graduate School of Letters, Kyoto University, Kyoto, Japan

**Keywords:** curiosity, responsiveness, middle childhood, late childhood, early childhood, parenting

## Abstract

Curiosity, the desire to learn new information, has a powerful effect on children’s learning. Parental interactions facilitate curiosity-driven behaviors in young children, such as self-exploration and question-asking, at a certain time. Furthermore, parenting quality predicts better academic outcomes. However, it is still unknown whether persistent parenting quality is related to children’s trait epistemic curiosity (EC). The current study examined whether parenting practices, responsiveness, and demandingness are cross-sectionally related to the trait EC of children in different age groups (preschoolers, younger and older school-aged children). We adopted a shortened Japanese version of the parenting style questionnaire and modified the trait EC questionnaire in young children. A sample of 244 caregivers (87.37% mothers) of children (ages 3–12) was recruited through educational institutions in Japan and reported on their parenting practices and trait EC. All data analyses were performed using SPSS version 26. Hierarchical regression analyses were performed to determine the explanatory variables for children’s trait EC. Self-reported parental responsiveness significantly explained EC scores. To the best of our knowledge, this is the first study to show a cross-sectional relationship between parental responsiveness and children’s trait EC. Future research should clarify whether parental responsiveness in early childhood predicts children’s EC later in life.

## Introduction

Curiosity is a fundamental learning motivation that involves the desire to know new or specific information (Berlyne, 1954, 1966). Epistemic curiosity (EC), one of the two branches of curiosity, is the drive not only to seek novel information but also to remove uncertainty (Berlyne, 1966; Loewenstein, 1994; Litman and Spielberger, 2003; Litman, 2005, 2008; Jirout and Klahr, 2012). Especially, experimental studies have shown that children’s active self-exploration occurs for the purpose of reducing uncertainty rather than merely seeking novel information (Schulz and Bonawitz, 2007; Cook et al., 2011; Blanco and Sloutsky, 2021). It has been suggested that EC is linked to well-being (Kashdan and Steger, 2007; Engel, 2009; Wang and Li, 2015) as well as academic achievement (Shah et al., 2018). Despite the positive aspects of EC, according to Engel (2009), children show less EC as they age, especially after starting school. Thus, supporting and maintaining children’s EC has long attracted caregivers and educators (Engel, 2011; Jirout, 2020).

EC was widely defined as a desire to seek various novelty stimuli and a specific knowledge or activity (Berlyne, 1954, 1960, 1966; Litman and Spielberger, 2003). According to Berlyne (1954, 1966), when humans encountered ambiguous stimuli, they experienced conflict. This experience motivates humans to explore new information to reduce this state (Berlyne, 1954, 1966). Litman and Spielberger (2003) theorized that individual differences exist in EC regarding the frequency of expressing EC. Building on this idea, Litman and Jimerson (2004) proposed that this personality trait is related to different emotions, pleasurable and aversive feelings of uncertainty, and pivotal aspects to motivate information seeking, and developed two types of EC model (Interest type EC/ Deprived type EC). Interest type EC (IEC) increases with the positive anticipation of learning new knowledge and motivates new information seeking (Litman, 2005). Deprived EC (DEC) increases undesirable feelings and motivates specific information seeking to reduce uncertainty (Litman, 2008). EC is the basic component of intrinsic motivation (Ryan and Deci, 2000), and IEC and DEC are related to different learning goals; as IEC is related to motivation to learn new knowledge simply for joy, while DEC is related to motivation to learn specific knowledge because of the “need to solve” (Litman and Jimerson, 2004). Specifically, DEC increases through extrinsic as well as intrinsic motivation (Litman et al., 2010).

Parenting is a factor that may affect children’s EC. Parenting style is defined as a persistent “constellation of attitudes toward the child that are communicated to the child and that, taken together, create an emotional climate in which the parent’s behaviors are expressed” (Darling and Steinberg, 1993, p. 488). Over the past decades, the impact of parenting style on child development has been investigated from two dimensions: responsiveness and demandingness (Baumrind, 1966; Maccoby and Martin, 1983). Responsiveness is referred to as the degree of warm acceptance with a sensitive response to children’s needs and interests (Landry et al., 2001, 2006). Demandingness refers to the degree of parental control with strictness in a child (Rodriguez et al., 2009). One of the major approaches to studying parenting styles is the typological approach. Parenting styles were classified into four categories based on this approach (Baumrind, 1967; Maccoby and Martin, 1983), which is one of the most adopted typology approaches (person-centered): authoritarian (low responsiveness, high demandingness), authoritative (high responsiveness, high demandingness), permissive (high responsiveness and low demandingness), and uninvolved/neglectful (low responsiveness and low demandingness). Another approach to studying parenting style is the dimensional approach (variable-centered), which examines the relationships between variables. This approach has been adopted to investigate parental practices (Power, 2013). In this study, we focus on a dimensional approach to examine the independent relationships of each parenting dimension.

The accumulated evidence indicates that parenting influences the development of children’s competence (for review, see Darling and Steinberg, 1993; Jeong et al., 2021). There has been empirical evidence of associations between responsiveness, demandingness, and cognitive development. For example, it has been noted that an authoritative parenting style, compared to authoritarian, permissive, and uninvolved/neglectful styles, has been positively associated with positive outcomes: academic achievement and cognitive development among adolescents (Steinberg et al., 1992; Llorca et al., 2017). Additionally, studies using the dimensional approach have also revealed a significant positive relationship between responsiveness and cognitive development, and academic success among elementary and middle school children (Dumont et al., 2014). A recent study has shown positive relations among parenting, which include parental encouragement and involvement in children’s school/home activities, learning motivation, and children’s EC among preschoolers (Kwok et al., 2022). Thus, warm and supportive parenting, such as high responsiveness, may guide children to achieve academic goals. However, existing evidence for the links between parenting and children’s EC is limited.

Parental support may play a major role in curiosity-driven behavior among children. Previous studies have revealed that a secure environment fosters children’s self-exploration in early childhood (Posada et al., 2007; Stupica et al., 2011), whereas an insecure environment is a key factor in infants’ poor exploration (Gaertner et al., 2008). Children’s information exploration is known to increase when they detect novelty (Berlyne, 1954, 1966), uncertainty (Schulz and Bonawitz, 2007; Blanco and Sloutsky, 2021), and knowledge gaps (Loewenstein, 1994; Stahl and Feigenson, 2015). Supporting children in recognizing EC-driven situations lead to higher levels of EC expression. For example, in an informal learning environment such as a museum, children aged three to seven actively engaged in complex material exploration when their parents guided them to do so. Conversely, children show less material exploration when parents actively explore tasks instead of guiding them to engage (Callanan et al., 2020). Furthermore, parental attitudes that encourage children to observe objects and ask questions at home increase complex exploration in preschoolers (Vandermaas-Peeler et al., 2019).

Based on these findings, the parent–child interactions over time are believed to be critical for the development of children’s trait EC. Trait EC is the individual difference in the frequency of curiosity-state experiences in general (Litman et al., 2005). This is a more persistent form of EC and is distinguished from curiosity-driven behavior at a particular moment (e.g., Naylor, 1981). It has been proposed that IEC, related to intrinsic motivation, is observable in infants without external encouragement to seek information (Oudeyer et al., 2016). However, consistent responsive parental behavior across infancy has been suggested to facilitate infants’ object exploration (Landry et al., 2003). Furthermore, responding to children’s interests and EC encouraged them to ask more questions and seek information (for review, see Torrance, 1966; Ronfard et al., 2018). A recent study showed that asking questions is a fundamental and important tool for children to express EC (Alaimi et al., 2020). Such parental practices may facilitate DEC expression, which involves extrinsic motivation such as reducing uncertainty or praise (Litman et al., 2010). Therefore, responsive parental practice may be related to an increase in opportunities for children to feel the desire to explore new objects (IEC) or detect ambiguity (DEC) in their daily lives. Specifically, responsive parental practices play an important role in creating a secure environment expressing IED and detecting uncertainty or information gap results in expressing DEC. However, although encouraging and responsive parental behavior facilitates children’s curiosity-driven behavior at a particular moment, little is known about whether responsive parenting is related to children’s trait EC.

Several studies have revealed that children’s EC expression changes from preschool to school age (Engel and Randall, 2009). It is commonly agreed that young children are naturally curious (Engel, 2009). Young children exhibit EC under more complex circumstances that require, for example, knowledge of cause-and-effect relationships in accordance with their cognitive maturity levels (FitzGibbon et al., 2019). However, in an observational study, researchers found that fifth-grade children showed less EC in the classroom than preschoolers (e.g., Engel, 2009). Engel (2009) pointed out that EC expression declines as people grow older after schooling. One possible interpretation for this is that the dominant goal of schools is mastery of skills rather than inquiry. Decreased EC levels in children may be partially related to school participation (Engel, 2009). Schools tend to provide fewer opportunities for children to express EC during late childhood (Engel, 2011). For instance, research has shown that in fifth-grade classrooms, educational priority focuses on mastering skills such as calculations and forms of grammar. This tendency has been observed in the early grades (Engel, 2011). Elementary school-aged children showed less exploration without the teacher’s permission to explore, especially girls (Coie, 1974). In the Japanese educational context, it has been suggested that elementary school teachers use a teacher-directed teaching approach (e.g., instruct declarative knowledge) when they consider the student’s need to master the targeted concept (Inoue et al., 2019), which may result in less opportunity for the student to show their EC at school. Additionally, a recent study has shown a significant relationship between parental school involvement, such as checking homework, and better academic outcomes among fourth-year students in Japan (Otani, 2020). The assumption is that EC might be generally high during early childhood, and children in this age group might have little room for an increase in EC because of parental influence. Therefore, the influence of parenting styles may be much greater for school-aged children than for preschoolers. Additionally, EC expression may be affected more by the school environment in older school-aged children; children reach adolescence when parental involvement declines (Steinberg, 2005).

Another view suggests that decreased levels of EC among school-aged children may be related to cognitive control development. Cognitive control enables children to regulate their behavior to achieve their goals. In general, school-aged children demonstrate better performance on cognitive control tasks than preschoolers (Davidson et al., 2006; Moriguchi and Hiraki, 2013; Chevalier et al., 2019; Plebanek and Sloutsky, 2019). Cognitive control abilities, such as executive function, enable older children to focus on goal-oriented information searches, which may be related to an increase in DEC expression. Simultaneously, cognitive control is also a predictor of better academic performance among elementary school students (Pascual et al., 2019). Academic activities or social academic expectations may influence whether older school-aged children express EC. Conversely, preschoolers performed better than older children on tasks such as remembering irrelevant information (Plebanek and Sloutsky, 2017; Gopnik, 2020; King and Markant, 2020; Blanco et al., 2023). One interpretation is that limited cognitive control encourages younger children to explore more information that is irrelevant to goals (Gopnik et al., 2017; Gopnik, 2020), which may be related to an increase in IEC expression.

In summary, parental support may facilitate the development of trait EC among children. Although several studies have focused on parental roles in encouraging children to explore new information at a certain time, the relationship between parenting and children’s EC remains poorly understood. Moreover, there is consistent evidence that preschool-aged children are more likely to express a higher EC than school-aged children. The EC in school-aged children dwindles as children get older. However, it is unclear whether the relationship with parenting differs between early and late childhood.

To the best of our knowledge, no studies have examined the relationship between parenting and children’s EC at different school levels. In the current study, we examined the relationship between parenting and trait EC in a sample of Japanese preschoolers and school-aged children. To this end, we classified children into three age groups (early, middle, and late childhood) because different educational and cognitive developmental levels may be related to children’s EC expression. To investigate whether parenting is related to children’s trait EC during different childhood periods, we analyzed the relationship between parenting and trait EC in preschoolers and younger and older school-aged children. This analytical approach is one of the main contributions of this study to the literature. We hypothesized that responsive parental practice would be related to children’s EC in middle childhood and would show fewer relationships in early and late childhood. We used a parent-reported-based questionnaire because it reflected children’s EC observed in a real-life setting (Piotrowski et al., 2014; Acar et al., 2019).

## Methods

### Participants

The participants were 245 caregivers of children aged 3–12 (girls 58.78%; mean age = 95.8 months; range 47–154 months; *SD* = 31.84). We determined the sample size based on the rule of thumb according to Harris’s formula for a correlation or regression sample size (VanVoorhis and Morgan, 2007). A sample of 10 to 30 participants per predictor variable was appropriate, or at least 50 plus the number of predictor variables (*N* > 54). In total, 245 parents answered questionnaires regarding their children’s EC and self-parenting styles. There was one case that was dropped from the analysis because of concerns about the validity of the data (*n* = 1). Based on the demographic questionnaire data completed by parents about themselves, the majority of caregivers described themselves as the child’s mother (87.37%). Details of the children’s current age in years and months and sex were provided. Participants’ children were divided into separate groups: preschoolers (*n* = 99, 55 girls, mean = 63.3 months, range = 47–83, *SD* = 10.18), younger school-aged (*n* = 72, 44 girls, mean = 99.5 months, range = 83–117, *SD* = 10.75), and older school-aged (*n* = 73, 45 girls, mean = 135.6 months, range = 118–154, *SD* = 10.47).

### Research design

A cross-sectional study was conducted to examine the relationship between parenting and children’s trait EC at different school levels. Participants were recruited from two Japanese educational institutions: a private elementary school that follows the Japanese national curriculum and a center for early childhood education under the jurisdiction of the Cabinet Office in mid-sized cities in Japan. All participants were Japanese speakers. Questionnaires were distributed to children by their teachers during January and February 2022. One of the main caregivers was asked to complete the questionnaire. Teachers distributed envelopes containing questionnaires to their students’ parents. Those who agreed to participate in the study returned a sealed, anonymous envelope to the children’s teachers. Before the study began, informed consent was obtained from each child’s caregiver. We included data when participants completed all items for at least one measure of children’s EC or self-parenting styles. All procedures were approved by the local psychological research ethics committee (3-P-23).

### Measures

#### Parenting style questionnaire

We used a parent-style questionnaire to investigate the quality of parental practices. Participants completed a shortened version of the Parenting Style Questionnaire (Robinson et al., 1995) developed in Japanese (Nakamichi and Nakazawa, 2003). Parenting style was assessed based on two dimensions: responsiveness and demandingness. Responses were recorded on a 4-point Likert scale (1 = *strongly disagree*, 4 = *strongly agree*). The responsiveness dimension scale was measured by eight items, for example: “When a child is playing alone and seems bored, join in and play with him or her” (one question was reverse scored) (Cronbach’s α = 0.79). The demandingness dimension was measured using eight items, for example, “Tell the child what to do” (two questions were reverse scored). For this study, we modified the demandingness scales by removing two statements that showed negative internal correlations in the factor of both items 12 and 14 (Cronbach’s α for the six demandingness items was 0.61). For more information, see Supplementary material S1.

#### Parent-reported epistemic curiosity

The caregivers completed the EC questionnaire (Piotrowski et al., 2014), which was translated into Japanese. In the questionnaire, five items assessed IEC, for example, “My child has fun learning about new topics or subjects” (Cronbach’s α = 0.88), and five items assessed DEC, for example, “When presented with a tough problem, my child focuses all of his/her attention on how to solve it” (Cronbach’s α = 0.90), using a 5-point Likert-type scale (1 = *almost never*, 5 = *almost always*). For more information, see Supplementary material S2.

### Data analysis

Since this study included a small sample size, determining the distribution of the variable parenting dimensions and children’s IEC/DEC was important for choosing an appropriate statistical method. Therefore, a Shapiro–Wilk test was performed, which showed that each variable deviated significantly from normality. Based on these results, a nonparametric test was used. The Kruskal–Wallis test was performed to examine group differences between the means of the three independent age groups. Spearman’s rank-order correlations were calculated to investigate the relationship between variables (sex, age in months, parenting styles, and EC) in this study for each age group. To examine whether parenting explained a statistical amount of variance in children’s IEC and DEC, a hierarchical regression analysis was performed. All data analyses were performed using SPSS version 26.

## Results

### Initial analysis

#### Descriptive and correlational analysis

Table 1 shows means and SD as well as comparisons *via* the Kruskal–Wallis test between the three different age groups. We did not find any significant group differences in IEC (Chi-square = 3.50, *p* = 0.174, *df* = 2) or DEC scores (Chi-square = 4.77, *p* = 0.09, *df* = 2). Table 2 illustrates the Spearman’s rank-order correlations between variables in each age group (preschoolers, younger school-aged, and older school-aged children) and across all children (age-collapsed). Responsiveness is positively related to DEC among preschoolers and EC (IEC and DEC) among younger-aged children. There were no significant relationships between parenting styles, responsiveness, demandingness, and children’s EC among older-aged children. The age-collapsed analysis showed weakened correlations between responsiveness and EC (IEC and DEC).

**Table 1:** Sample descriptive separately for the three age groups.

	Total (*n* = 244)		Preschoolers (*n* = 99)		Younger school-age (*n* = 72)		Older school-age (*n* = 73)		Group differences	
	*n*	*M* (SD)	*n*	*M* (SD)	*n*	*M* (SD)	*n*	*M* (SD)	Value of *p*	Kruskal-Walls Test
Responsiveness	241	3.00 (0.50)	97	2.89 (0.49)	72	3.10 (0.52)	72	3.06 (0.45)	0.018	Yo > Pr
Demandingness	237	3.38 (0.39)	95	3.22 (0.39)	70	3.45 (0.34)	72	3.50 (0.38)	0.000	Yo > Pr, Ol > Pr
IEC	240	4.38 (0.65)	97	4.41 (0.68)	72	4.46 (0.50)	71	4.25 (0.72)	0.174	
DEC	241	3.61 (0.84)	98	3.72 (0.77)	71	3.66 (0.81)	72	3.43 (0.93)	0.092	

Higher scores indicate higher parenting practices of each dimension. Higher scores indicate more curious. IEC, I-type epistemic curiosity; DEC, D-type epistemic curiosity; Pr, preschoolers; Yo, younger school-age; Ol, older school-age.

**Table 2:** Spearman’s rank order correlations between variables.

	1	2	3	4	5
All (*n* = 244)					
1. Sex	–				
2. Age in months	0.05	–			
3. Responsiveness	−0.03	0.10	–		
4. Demandingness	−0.10	0.29**	0.23**	–	
5. IEC	0.00	−0.10	0.22**	0.07	—
6. DEC	−0.02	−0.12	0.19**	0.01	0.70**
Preschoolers (*n* = 99)					
1. Sex	—				
2. Age in months	0.01	–			
3. Responsiveness	0.06	−0.17	—		
4. Demandingness	0.04	0.06	0.34**	–	
5. IEC	−0.07	0.10	0.08	0.19	–
6. DEC	−0.03	0.00	0.23*	0.09	0.66**
Younger school-age (*n* = 72)					
1. Sex	–				
2. Age in months	0.11	–			
3. Responsiveness	−0.25*	−0.17	—		
4. Demandingness	−0.13	−0.15	0.07	—	
5. IEC	−0.13	−0.05	0.50**	0.24*	—
6. DEC	−0.16	−0.02	0.51**	0.14	0.70**
Older school-age (*n* = 73)					
1. Sex	—				
2. Age in months	0.05	—			
3. Responsiveness	0.12	0.00	—		
4. Demandingness	−0.27*	0.11	0.13	—	
5. IEC	0.22	−0.17	0.14	−0.09	—
6. DEC	0.12	−0.03	−0.08	−0.04	0.69**

∗*p* < 0.05, ∗∗*p* < 0.01.

#### Relationship between parenting styles and children’s curiosity

A hierarchical regression analysis was performed to determine whether parenting practices are a significant predictor of children’s IEC and DEC beyond their age. A two-step hierarchical multiple regression analysis was performed with each of the children’s IEC and DEC as the dependent variables. Children’s sex was entered in Step 1, and their age, parenting dimensions, responsiveness, and demandingness were entered in Step 2 as predictors. For all children, children’s sex, age, and parenting dimensions did not contribute significantly to the regression model IEC [*F*(4,224) = 0.41, *p* = 0.119]. In contrast, Children’s sex, age, and parenting dimensions contributed significantly to the regression model DEC [*F*(4,225) = 3.37, *p* = 0.011]. Furthermore, we found that parental responsiveness significantly explained IEC score, and children’s age and parental responsiveness significantly explained DEC score. These results indicated that children’s IEC and DEC were higher with increasing levels of responsive parental practice and children’s DEC decreases as they mature. For preschoolers, multiple regression analysis revealed that at Stage 2, children’s sex, age, and parenting dimensions did not contribute significantly to the regression models IEC [*F*(4,84) = 1.40, *p* = 0.242] or DEC [*F*(4,85) = 1.09, *p* = 0.366]. We found that children’s age explained IEC, indicating that preschoolers’ IEC increases as they get older. For younger-aged children, children’s sex, age, and parenting dimensions contributed significantly to the regression models IEC [*F*(4,65) = 5.17, *p* < 0.001] and DEC [*F*(4,64) = 5.04, *p* < 0.001]. Furthermore, we found that parental responsiveness significantly explained IEC and DEC scores. For younger-aged children, ECs were higher with increasing levels of responsive parental practice. For older school-aged children, children’s sex, age, and parenting dimensions did not contribute significantly to the regression models IEC [*F*(4,65) = 1.86, *p* = 0.128] or DEC [*F*(4,66) = 0.36, *p* = 0.838] (Table 3).

**Table 3:** Hierarchical regression analysis for variables predicting IEC and DEC scores.

	All			Preschoolers			Younger school-age			Older school-age		
	B	SE B	β	B	SE B	β	B	SE	β	B	SE	β
IEC												
Step 1												
Child’s sex	0.027	0.087	0.021	−0.056	0.146	−0.041	−0.105	0.123	−0.103	0.278	0.176	0.188
R2	0.000			0.002			0.011			0.035		
Step2												
Child’s sex	0.044	0.087	0.034	−0.072	0.144	−0.053	0.042	0.115	0.041	0.259	0.182	0.176
Child’s age	−0.003^†^	0.001	−0.126	0.014*	0.007	0.222	0.002	0.005	0.033	−0.015^†^	0.008	−0.223
Responsiveness	0.183*	0.088	0.141	0.039	0.154	0.028	0.447**	0.109	0.468	0.026	0.192	0.016
Demandingness	0.003	0.116	0.002	0.165	0.194	0.095	0.232	0.162	0.159	−0.207	0.232	−0.111
R2	0.032			0.062			0.241**			0.103		
												
DEC												
Step 1												
Child’s sex	−0.085	0.113	−0.049	−0.122	0.168	−0.078	−0.275	0.196	−0.169	0.178	0.226	0.094
R2	0.002			0.006			0.029			0.009		
Step2												
Child’s sex	−0.056	0.112	−0.032	−0.127	0.167	−0.081	−0.068	0.185	−0.042	0.220	0.242	0.116
Child’s age	−0.005*	0.002	−0.172	0.006	0.008	0.084	0.002	0.008	0.033	−0.004	0.011	−0.046
Responsiveness	0.303**	0.113	0.179	0.308^†^	0.180	0.193	0.726**	0.176	0.470	−0.214	0.253	−0.105
Demandingness	0.024	0.149	0.011	0.038	0.227	0.019	0.168	0.260	0.072	0.070	0.310	0.029
R2	0.056*			0.049			0.240**			0.021		

^†^*p* < 0.10. ^∗^*P* < 0.05. ^∗∗^*P* < 0.01. B, partial regression coefficient; SE, standard error; β, standardized partial regression coefficient.

## Discussion

In the present study, we investigated whether responsive and demanding parental practices are related to children’s trait EC during early, middle, and late childhood in a sample of Japanese children. The results showed that responsive parenting was related to children’s trait EC. In addition, parents who exhibited acceptance and warm behavior were more likely to have children who expressed higher levels of IEC and DEC, especially during middle childhood. However, parental practices were not significantly related to trait EC in older school-aged children (9-to 12-year-olds). We found no significant relationship between sex and trait EC in any age group. This is partly consistent with a previous study that reported that the trait EC of 3-to 8-year-old children was not significantly different between the sexes (Piotrowski et al., 2014).

Our results indicate that parental responsive involvement was related to trait EC, both IEC and DEC. Inconsistent with the evidence that school-aged children showed less EC than preschoolers (Engel, 2011), our data indicate that there are no significant EC differences between children. This may be because the present study reflected children’s EC observed at home, whereas the previous study reflected children’s EC observed in a school setting. Our findings could imply that responsive parenting from preschool age through middle childhood may promote children’s EC. Previous studies have reported that parental responsive interactions during early childhood predict children’s social or cognitive abilities in later childhood, including prosocial behavior (Stern and Cassidy, 2018), cognitive skills (Hurtado-Mazeyra et al., 2022), and math achievement (Duncan et al., 2019). Responsive parenting has also been linked to parental involvement in schools. These studies indicate that parental school involvement could be a moderator in predicting future outcomes in children. Previous studies have also suggested that parental beliefs influence children’s attitudes and interests (Leibham et al., 2005; Cevher-Kalburan and Ivrendi, 2016; Pattison and Dierking, 2019). When parents believed that curiosity was important for their children’s future, they tended to provide more opportunities related to their children’s interests (Leibham et al., 2005). In contrast, demanding-only parenting may be related to less encouragement of children’s interests (LaForett and Mendez, 2017). Taken together, we propose that parental responsiveness facilitates children’s EC development.

When we looked more closely, no significant relationship was detected between parenting practices and trait EC among older children. One possible interpretation is that older school-aged children’s EC levels may be affected by other factors such as school-related activities as children reach a period of becoming more independent from their parents (Feder et al., 2019). Furthermore, cognitive control skills enable older children to focus on goal-related activities. The influence of the school context increases when children in late childhood place greater importance on academic activities. Another possibility is that parents who show responsive parenting may focus on their children’s academic achievement during late childhood. Indeed, responsiveness has been reported to be related to parental involvement in education during late childhood and to be a predictor of better academic outcomes among adolescents (Dumont et al., 2014). In the Japanese educational context, teachers tend to teach knowledge to master skills instead of adopting a student inquiry approach (Henry and Brown, 2008; Inoue et al., 2019). This may reflect that the standardized entrance exam is a central determinant of acceptance into higher education in Japan (Yamamoto and Brinton, 2010). Responsive parents may consider the importance of academic success involving academically related activities when their children get older. However, it is important to consider both EC development and knowledge enhancement. Importantly, a recent longitudinal study indicated that 8-to 10-year-old children’s curiosity predicted longer-term curiosity characteristics (Fandakova et al., 2018). Middle and late childhood can be critical ages for fostering a later curious mind. Thus, further research is required to understand whether the dominance of mastering skills affects children’s curiosity development as they get older. Previous studies have revealed that encouraging parental interaction promotes object exploration in children at a certain age (Posada et al., 2007; Stupica et al., 2011; Vandermaas-Peeler et al., 2019; Callanan et al., 2020). We investigated a more sustained relationship between parent–child interaction and EC development. Our results showed that responsive parenting was cross-sectionally related to trait EC among, especially, younger school-aged children. However, the relationship with parenting did not persist in later childhood. Previous studies have indicated that the parenting combination of responsiveness and demandingness (authoritative) parenting is positively correlated to children’s academic achievement (Burchinal et al., 2002) and school adjustment (Chen et al., 1997). A key feature of our findings is that school activity may have a greater influence on children’s EC development than parental involvement in late childhood. Furthermore, parenting may also influence school-related tasks that are prioritized in the educational system.

### Implication

The main aim of the current study was to address whether parenting practices are linked to the trait EC of children in different age groups. While previous research has focused on parental roles in enhancing children’s EC at a certain age, our results show that responsive parenting is cross-sectionally related to children’s trait EC in middle childhood. Responsive parenting may be beneficial for curiosity development among children, especially in middle childhood. Future work could examine the relationship between the educational approach (knowledge enhancement or inquiry) and children’s EC.

### Limitation

The current study had several limitations. First, cross-sectional correlations do not determine whether parental responsiveness influences EC development. Longitudinal studies are needed to examine whether (a) EC changes during childhood and (b) parental responsiveness at an early stage of life predict children’s EC development in later childhood. Second, we did not investigate educational expectations or beliefs about the importance of curiosity among parents and teachers. Further research is needed to address how social and educational expectations affect children’s EC, especially during late childhood. Specifically, it will be of interest to examine how parental beliefs affect children’s EC development over the long term. Additional research is needed to examine the relationships between children’s IEC and DEC, cognitive control ability, teaching approach (e.g., inquiry-based teaching or teacher-direct teaching), and parenting style in different age groups.

### Conclusion

Our findings shed new light on the importance of a responsive parental attitude for fostering and maintaining trait EC in middle childhood. Our results showed no significant association between parenting practices and children’s trait EC during early and late childhood. These findings suggest the contribution of other factors, such as cognitive status and school activities, to curiosity. This study offers some guidance for future investigations of factors predicting children’s curiosity. Future research should clarify whether parental responsiveness at an early age plays a vital role in EC in later childhood when children engage in school activities.

## Data availability statement

The datasets presented in this study can be found in online repositories. The names of the repository/repositories and accession number(s) can be found at: https://osf.io/p2utz/?view_only=2ef5c25bbe2a4e268b4d3f0cbe1a5de1.

## Ethics statement

The studies involving human participants were reviewed and approved by Kyoto University Psychological Science Unit. The participants provided their written informed consent to participate in this study.

## Author contributions

SI contributed to the conceptualization of the study based on suggestions made by YM and KS, collected the data, conducted the analyses, and drafted the original manuscript. All authors critically reviewed and revised the manuscript draft and approved the final version for submission.

## Funding

This work was supported by JST SPRING, grant number JPMJSP2110.

## Conflict of interest

The authors declare that the research was conducted in the absence of any commercial or financial relationships that could be construed as a potential conflict of interest.

## Publisher’s note

All claims expressed in this article are solely those of the authors and do not necessarily represent those of their affiliated organizations, or those of the publisher, the editors and the reviewers. Any product that may be evaluated in this article, or claim that may be made by its manufacturer, is not guaranteed or endorsed by the publisher.
