# Supplementary material for: Parental responsiveness and children’s trait epistemic curiosity
Source: Front Psychol. 2023 Jan 26;13:1075489. doi: 10.3389/fpsyg.2022.1075489 (PMC9910790; doi:10.3389/fpsyg.2022.1075489)
Supplement: Supplementary file 1 [file Table_1.DOCX]

Supplementary Material

**S1**

Concerning the parental styles scale, we used a shortened version of parenting style questionnaire (Robinson, Mandleco, Olsen, & Hart, 1995) developed in Japanese (Nakamichi & Nakazawa, 2003). In the current study, we modified this scale and deleted two items (item12 and item14).

**Table S1.** *Items on Parenting Style Questionnaire (Responsiveness1-8, Demandingness 9-16)*

| Item | |
| --- | --- |
| 1 | When a child is playing alone and seems bored, join in and play with him or her. |
| 2 | Showing affection by hugging your child and saying kind words to him or her. |
| 3 | When you think your child is frustrated, ask him or her what's wrong. |
| 4 | Spend time with your child when you are home, playing ball, games, etc. |
| 5 | When you are out somewhere and your child feels tired, rest or hold your child. |
| 6 | When you are busy and your child wants to play, you play together. |
| 7 | When your child misbehaves, ask them why they did it and discuss what you should have done. |
| 8 | When you go on family playdates, incorporate as much as possible where the child wants to go, not just at the parent's convenience. |
| 9 | When your child does not keep a promise, he/she made to you, reiterate the promise to him/her. |
| 10 | Force your child to be quiet in places where quiet is required, such as libraries and movie theaters |
| 11 | When your child does not do what he or she is supposed to do, say, "Do it. |
| 12 | When you go shopping and don't plan to buy a toy, but your child wants a toy and won't move from the store, don't buy the toy. |
| 13 | When your child is playing with a friend and takes a toy that the friend is using, and you force him or her to give it back. |
| 14 | When your child is playing and not going to bed at bedtime, you force sleep. |
| 15 | When your child is making a fuss because something he or she is doing is not working, quiet him or her down. |
| 16 | Tell not to use bad language or talk to your child if he/she uses bad language with you ("stupid," "jerk," etc.). |

*Note.* For the current study, items 12 and 14 are removed because they show negative internal correlations.

**S2**

Concerning the children’s curiosity scale, we used a translated version of the epistemic curiosity questionnaire (Piotrowski, Litman, & Valkenburg, 2014).

**Table S2.** *Items on Epistemic Curiosity Questionnaires (Interest type curiosity 1-5, Deprived type curiosity 6-10)*

| Item | |
| --- | --- |
| 1 | My child has fun learning about new topics or subjects. |
| 2 | My child is attracted to new things in his/her environment. |
| 3 | My child enjoys talking about topics that are new to him/her. |
| 4 | My child shows visible enjoyment when discovering something new. |
| 5 | When my child is learning something new, he/she asks many questions about it. |
| 6 | When presented with a tough problem, my child focuses all his/her attention on how to solve it. |
| 7 | My child devoted considerable effort trying to figure out things that are consuming or unclear. |
| 8 | My child bothered when he/she does not understand something and tries hard to make sense of it. |
| 9 | My child will work for a long time to solve a problem because he/she wants to know the answer. |
| 10 | My child carefully examines things by turning them around or looking at them from all sides. |

**
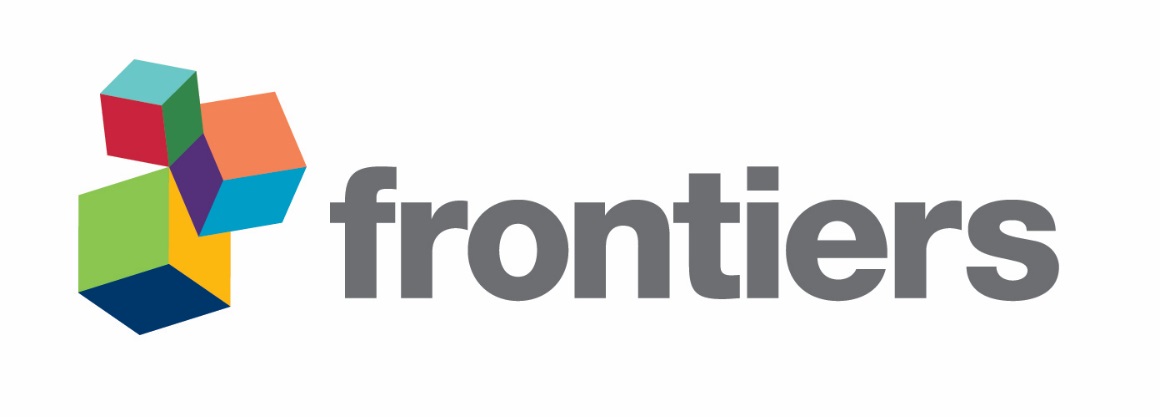
**
